# Impact of erroneous meal insulin bolus with dual-hormone artificial pancreas using a simplified bolus strategy - A randomized controlled trial

**DOI:** 10.1038/s41598-018-20785-4

**Published:** 2018-02-08

**Authors:** Véronique Gingras, Mohamed Raef Smaoui, Charlotte Cameli, Virginie Messier, Martin Ladouceur, Laurent Legault, Rémi Rabasa-Lhoret

**Affiliations:** 1 Institut de Recherches Cliniques de Montréal, Montreal, Quebec Canada; 2 Department of nutrition, Université de Montréal, Montreal, Quebec Canada; 3 Université de Rennes 1, Rennes, France; 4 Research Center of the Université de Montréal Hospital Center (CRCHUM), Montreal, Quebec Canada; 5 Montreal Children’s Hospital, McGill University Health Center, Montreal, Quebec Canada; 6 Montreal Diabetes Research Center (MDRC), Montreal, Quebec Canada

## Abstract

Postprandial glucose control remains challenging for patients with type 1 diabetes (T1D). A simplified meal bolus approach with a dual-hormone (insulin and glucagon) closed-loop system (DH-CLS) has been tested; yet, the impact of categorization errors with this strategy is unknown. The objective was to compare, in a randomized controlled inpatient trial, DH-CLS with the simplified meal bolus approach for two different meals properly categorized or overestimated. We tested, in patients with T1D, the simplified strategy with two standardized breakfasts (n = 10 per meal) adequately categorized or overestimated: (1) 75 g and (2) 45 g of carbohydrate. No difference was observed for percentage of time <4.0 mmol/L over a 4-hour post-meal period (primary outcome; median [IQR]: 0[0–0] vs. 0[0–0] for both comparisons, p = 0.47 and 0.31 for the 75 g and 45 g meals, respectively). Despite higher meal insulin boluses with overestimation for both meals (9.2 [8.2–9.6] vs. 8.1 [7.3–9.1] U and 8.4 [7.2–10.4] vs. 4.8 [3.7–5.6] U; p < 0.05), mean glycemia, percentage of time in target range and glucagon infusion did not differ. Additional scenarios were tested *in silico* with comparable results. These results suggest that the DH-CLS with a simplified meal bolus calculation is probably able to avoid hypoglycemia in the event of meal size misclassification.

## Introduction

The closed-loop delivery systems for glucose control in type 1 diabetes could ease some burden associated with treatment for type 1 diabetes patients. Compared to continuous subcutaneous insulin infusion, these systems are improving glucose control and reducing the risk of hypoglycemia significantly^1–4^. Yet, even in the context of closed-loop delivery, postprandial glucose control remains a challenge^5^. With intensive insulin therapy for type 1 diabetes, patients need to estimate the carbohydrate content of their meals and determine the proper insulin bolus based on their insulin-to-carbohydrate ratio^6^. In a closed-loop strategy, the subcutaneous infusion rate is modulated based on recommendations generated by an algorithm and relying on continuous glucose monitoring sensor readings^7^. With this strategy, several attempts have been made to alleviate entirely^8,9^ or simplify meal insulin boluses^10,11^. We have developed a simplified meal bolus strategy for the closed-loop delivery system based on a semi-quantitative assessment of the meal carbohydrate content. In this strategy, patients choose a meal category between three options (regular, large or very large corresponding respectively to 30–60 g, 60–90 g and >90 g of carbohydrate) and receive a meal bolus based on a fixed carbohydrate factor for each category (35 g, 65 g and 95 g, respectively), multiplied by their individual insulin-to-carbohydrate ratio^11,12^. In previous studies, this strategy yielded similar mean glucose and time spent in target range compared to the carbohydrate-matched boluses; yet, adjustments remained needed to mitigate the risk of hypoglycemia^11,12^. This strategy thus has the potential to reduce the burden associated with precise carbohydrate counting, which is challenging for patients^13^, without degrading glucose control. However, errors in carbohydrate counting are frequent^14^ and with this simplified semi-quantitative strategy, misclassification of the meal could result in a large overestimation or underestimation of the meal bolus. It is thus important to establish the potential safety of this simplified meal bolus approach in the context of meal misclassification.

The main objective of the present study is to examine the impact of overestimating a meal insulin bolus (erroneous categorization) in the context of dual-hormone closed-loop delivery with a simplified meal bolus strategy in adult patients with type 1 diabetes. Dual-hormone closed-loop delivery was chosen to limit the potential hypoglycemia risk associated with overestimation of meal insulin boluses. This study is a single-blind, randomized, two-way, cross-over study to compare 1) Dual-hormone closed-loop delivery with a simplified meal insulin bolus strategy and an adequately estimated (proper categorization) meal size bolus, 2) Dual-hormone closed-loop delivery with a simplified meal insulin bolus strategy and with an overestimated (erroneous categorization) meal size bolus. Two meal scenarios were tested in clinical settings during two consecutive clinical trials: 1) a 75 g of carbohydrate meal either adequately estimated (bolus for 65 g of carbohydrate) or overestimated (bolus for 95 g of carbohydrate) and 2) a 45 g of carbohydrate meal either adequately estimated (bolus for 35 g of carbohydrate) or overestimated (bolus for 65 g of carbohydrate). We hypothesized that dual-hormone closed-loop delivery with overestimated meal size insulin bolus will not increase time below 4.0 mmol/L compared to dual-hormone closed-loop delivery with an adequately estimated meal size insulin bolus. Furthermore, to provide additional insight into the clinical outcomes related to the simplified meal bolus strategy, we simulated *in silico* similar scenarios along with a 105 g of carbohydrate meal with an underestimated, adequately estimated, and overestimated insulin bolus.

## Results

**Figure 1:**
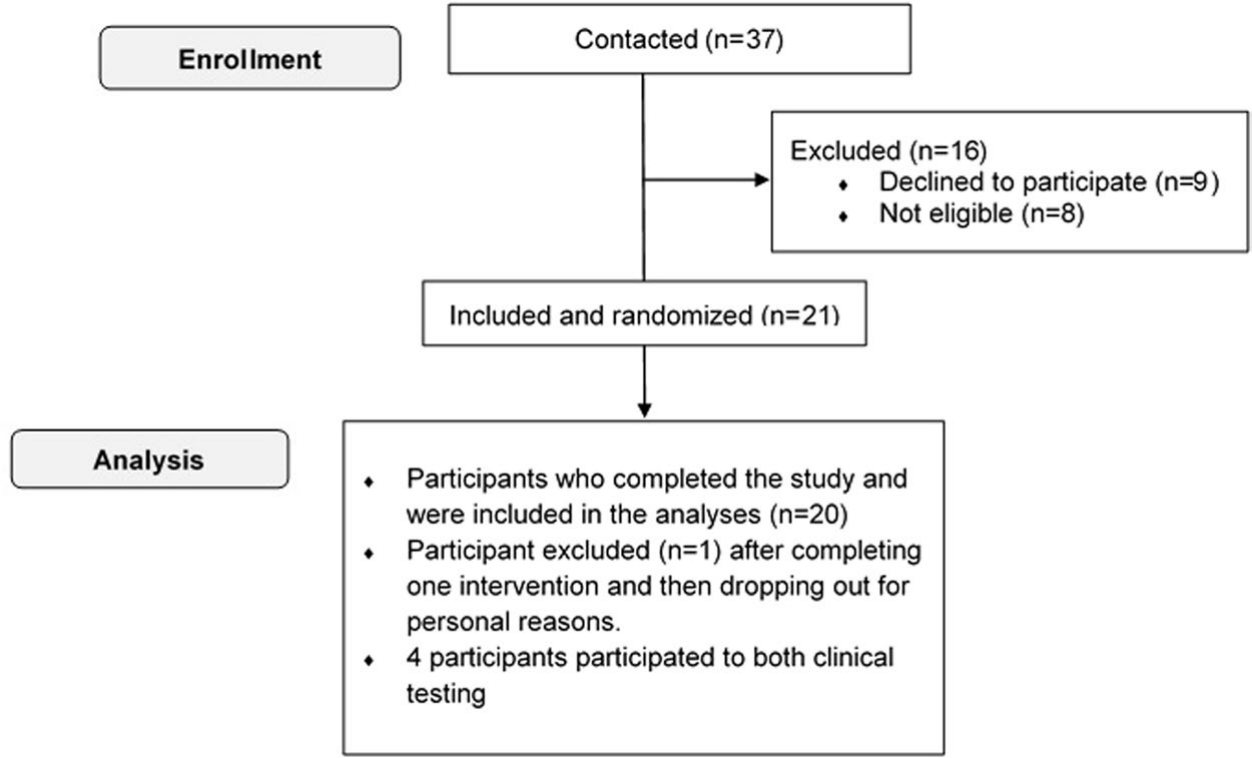
Flow of participants in the clinical trials.

Among the twenty-one participants included and randomized in this study for the clinical trials (Fig. 1), twenty (ten per meal) completed both interventions and were included in the analysis. Participants (11 Women – 9 Men) had a mean glycated hemoglobin of 7.3 ± 0.5% and 7.3 ± 0.8%, as well as a duration of diabetes of 23.3 ± 14.9 and 24.5 ± 13.4 years for the 75 g and 45 g of carbohydrate meals, respectively (Table 1). Characteristics of the participants in the virtual trials are presented in Table 2.

**Table 1:** Characteristics of participants in real clinical trials.

Characteristics	75 g of carbohydrate meal (n = 10)	45 g of carbohydrate meal (n = 10)
	Median [IQR]	Median [IQR]
Age, years	39.0 [24.3–55.8]	30.5 [24.3–49.8]
Duration of diabetes, years	23.0 [11.0–34.3]	24.5 [12.5–35.0]
Body weight, kg	70.5 [63.8–88.5]	70.2 [61.3–83.9]
Body mass index, kg/m^2^	25.0 [22.7–27.6]	25.3 [22.7–26.8]
Glycated hemoglobin, %	7.2 [6.9–7.5]	7.3 [7.1–7.6]
Total daily basal insulin dose (U/day)	22.4 [16.8–26.3]	24.9 [17.1–29.8]
Total daily insulin dose (U/day)	43.5 [37.0–45.7]	47.0 [41.7–52.8]

**Table 2:** Characteristics of participants in virtual clinical trials.

Characteristics	Median [IQR]
Age, years	47.0 [38.8–52.5]
Duration of diabetes, years	28.0 [8.0–55.0]
Body weight, kg	67.2 [57.0–80.2]
Body mass index, kg/m^2^	26.0 [19.0–35.0]
Glycated hemoglobin, %	8.0 [7.0–9.0]
Total daily basal insulin dose (U/day)	42.9 [23.2–62.0]

**Figure 2:**
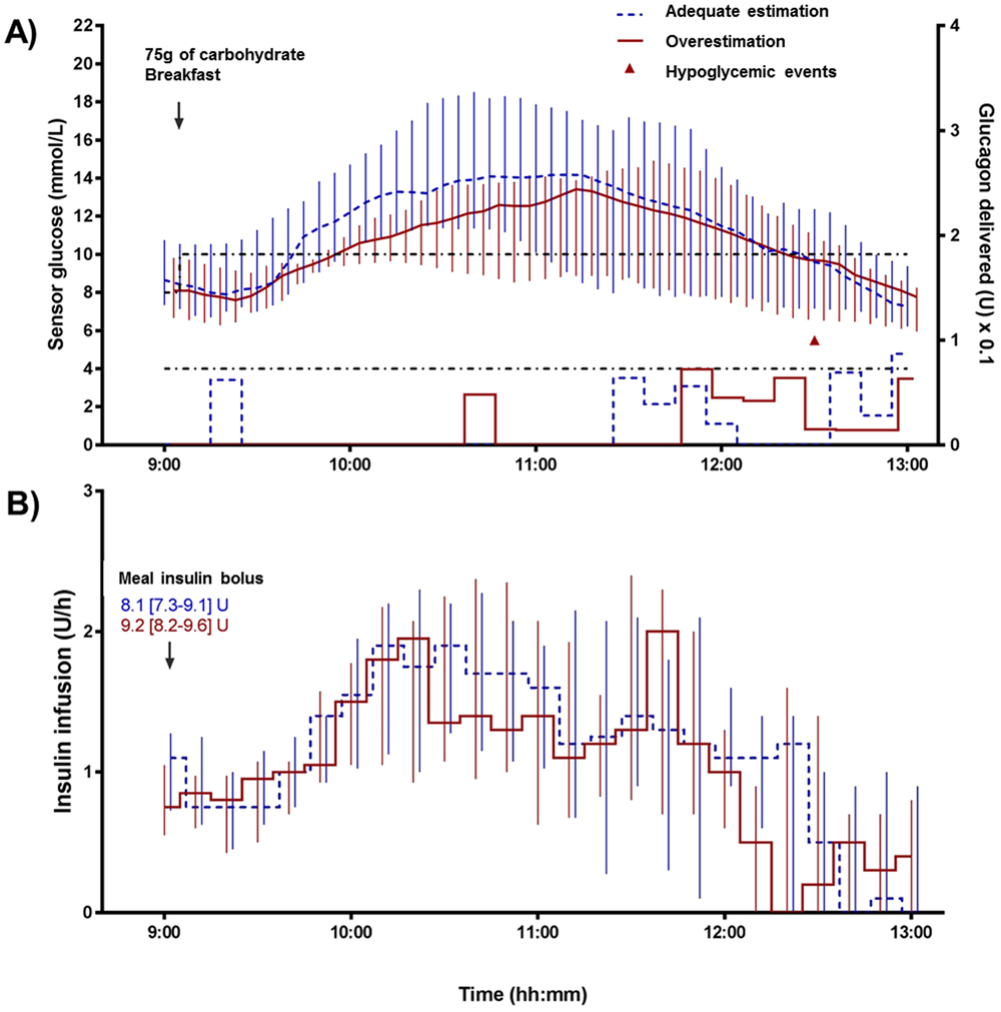
Profile (median and interquartile ranges) of sensor glucose, hypoglycemic events and glucagon infusion (**A**) as well as insulin infusion and boluses (**B**) during the 4-h postprandial period following the 75 g of carbohydrate meal in the clinical trial.

**Figure 3:**
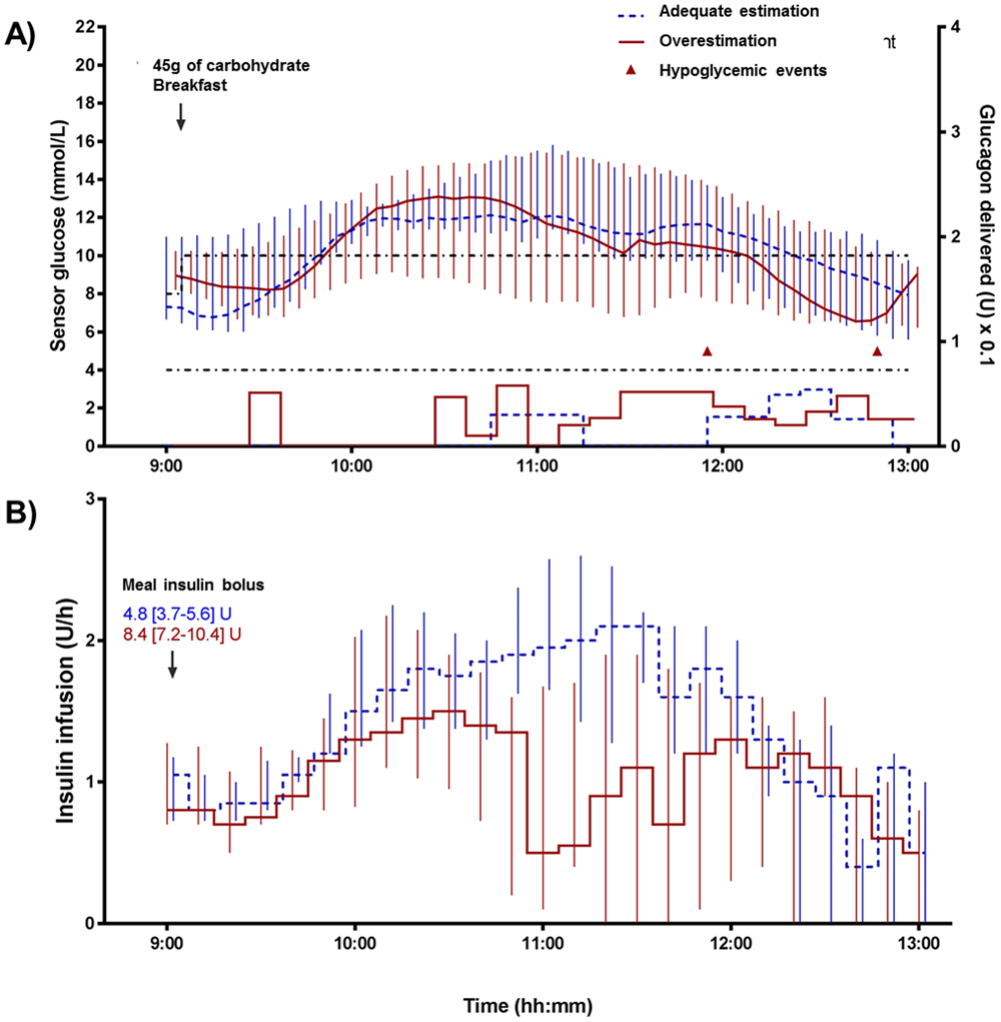
Profile (median and interquartile ranges) of sensor glucose, hypoglycemic events and glucagon infusion (**A**) as well as insulin infusion and boluses (**B**) during the 4-h postprandial period following the 45 g of carbohydrate meal in the clinical trial.

Figures 2 and 3 show the profile (median and interquartile ranges) of sensor glucose, hypoglycemic events, glucagon infusion as well as insulin infusion and boluses during the 4-h period following both meals. The primary outcome of this study was the percentage of time below 4.0 mmol/L (Table 3). For both meals, percentage of time <4.0 mmol/L did not differ between the adequate estimation and overestimation (0 [0–0] vs. 0 [0–0]% for both meals, p = 0.47 and p = 0.31 for the 75 g and 45 g meals, respectively). The same outcome was observed for the virtual trials in Table 4. In total, three episodes of hypoglycemia were observed, all with the overestimation of the meal bolus (1 in the 75 g meal and 2 in the 45 g meal), and all episodes occurred in the late postprandial period (between 3 and 4-h post-meal). For both the 75 g and the 45 g of carbohydrate meal trials, mean sensor glucose in the 4-h post-meal period did not differ (11.1 [8.6–13.6] vs. 10.2 [8.0–11.9]; p = 0.18 and 10.3 [9.0–12.2] vs. 9.7 [7.5–13.1]; p = 0.21) between adequate estimation and overestimation; yet, a high percentage of time in hyperglycemia was observed (69 [29–89] vs. 54 [9–72]; p = 0.29 and 54 [40–94] vs. 44 [6–90]; p = 0.14) in all interventions. No significant difference was observed in any of the other secondary outcomes including time in target range (4.0 to 10.0 mmol/L), peak glucose, and time to peak glucose between overestimation or adequate estimation (p > 0.05).

**Table 3:** Comparison of outcomes among adults with type 1 diabetes in real clinical trials.

Outcomes	75 g carbohydrate meal (n = 10)			45 g carbohydrate meal (n = 10)		
	Adequate carbohydrate estimation	Overestimated carbohydrate content	P-Value	Adequate carbohydrate estimation	Overestimated carbohydrate content	P-Value
	Median [IQR]	Median [IQR]		Median [IQR]	Median [IQR]	
% Time <4.0 mmol/L	0 [0–0]	0 [0–0]	0.47	0 [0–0]	0 [0–0]	0.31
Hypoglycemic events	0	1	—	0	2	—
Starting glucose, mmol/L	8.7 [7.3–10.8]	8.0 [6.7–9.8]	0.48	7.3 [6.7–11.0]	9.0 [8.2–10.3]	0.66
Mean glucose, mmol\L	11.1 [8.6–13.6]	10.2 [8.0–11.9]	0.18	10.3 [9.0–12.2]	9.7 [7.5–13.1]	0.21
% Time 4–10 mmol/L	31 [15–71]	45 [28–88]	0.44	46 [6–60]	53 [10–76]	0.22
% Time >10.0 mmol/L	69 [29–89]	54 [9–72]	0.29	54 [40–94]	44 [6–90]	0.14
Peak glucose, mmol/L	14.6 [11.8–18.7]	13.3 [10.2–14.9]	0.15	13.7 [11.8–18.7]	13.7 [9.8–15.7]	0.15
Time to peak, minutes	93 [70–114]	125 [78–154]	0.17	108 [95–120]	80 [70–116]	0.72
Insulin bolus, units	8.1 [7.3–9.1]	9.2 [8.2–9.6]	0.03	4.8 [3.7–5.6]	8.4 [7.2–10.4]	<0.001
Basal insulin infusion, units	4.2 [3.9–5.3]	4.3 [3.1–5.4]	0.60	5.7 [4.1–7.4]	4.3 [2.8–5.2]	0.06
Glucagon delivery, mg	2.9 [0–5.8]	1.0 [0–4.5]	0.91	0 [0–4.8]	0.5 [0–5.9]	0.55

**Table 4:** Comparison of outcomes among adults with type 1 diabetes in virtual clinical trials.

Outcomes	105 g carbohydrate meal (n = 15)			75 g carbohydrate meal (n = 15)			45 g carbohydrate meal (n = 15)	
	Underestimated carbohydrate content	Adequate carbohydrate estimation	Overestimated carbohydrate content	Underestimated carbohydrate content	Adequate carbohydrate estimation	Overestimated carbohydrate content	Adequate carbohydrate estimation	Overestimated carbohydrate content
	Median [IQR]	Median [IQR]	Median [IQR]	Median [IQR]	Median [IQR]	Median [IQR]	Median [IQR]	Median [IQR]
% Time <4.0 mmol/L	0 [0–0]	0 [0–0]	0 [0–0]	0 [0–0]	0 [0–0]	0 [0–0]	0 [0–0]	0 [0–0]
Starting glucose, mmol/L	5.3 [4.8–6.4]	5.1 [4.8–6.3]	5.3 [4.9–6.3]	5.4 [4.9–6.1]	5.5 [4.8–6.1]	5.3 [5.0–6.1]	5.3 [4.9–6.1]	5.3 [5.0–6.3]
Mean glucose, mmol\L	11.7 [8.9–13.3]	10.1 [7.1–11.9]	9.3 [7.2–12.1]	11.9 [9.8–12.6]	9.1 [6.8–10.1]	6.8 [6.1–10.6]	9.3 [7.9–10.2]	7.0 [5.5–8.3]
% Time 4–10 mmol/L	36 [22–54]	40 [28–72]	44 [28–68]	32 [22–44]	52 [42–92]	52 [38–100]	56 [42–78]	84 [50–100]
% Time >10.0 mmol/L	64 [38–74]	60 [10–72]	40 [0–70]	68 [46–78]	48 [4–54]	0 [0–60]	44 [18–58]	0 [0–20]
Peak glucose, mmol/L	17.5 [12.1–20.0]	13.9 [9.3–18.1]	13.7 [8.8–17.2]	15.9 [15.2–18.1]	12.3 [9.2–13.3]	8.9 [7.8–13.6]	11.6 [10.3–14.1]	9.3 [8.0–11.1]
Time to peak, minutes	110 [90–155]	130 [80–190]	90 [70–125]	120 [90–150]	100 [70–180]	110 [80–145]	100 [85–145]	80 [55–90]
Insulin bolus, units	5.8 [5.0–6.4]	8.1 [6.5–9.1]	8.7 [7.9–10.5]	2.9 [2.5–3.4]	5.7 [5.0–6.2]	8.1 [6.5–8.9]	2.9 [2.5–3.3]	5.7 [5.0–6.4]
Basal insulin infusion, units	36.6 [19.8–46.4]	29.7 [13.5–42.9]	23.6 [12.1–39.3]	35.6 [27.0–45.2]	23.1 [12.6–40.1]	18.1 [12.6–32.7]	30.9 [17.1–36.1]	12.0 [8.5–29.0]
Glucagon delivery, mg	0 [0–4.1]	0 [0–11.1]	1.8 [0–4.7]	0 [0–1.4]	0 [0–0.8]	0 [0–3.7]	0 [0–3.6]	0 [0–12.9]

For the 75 g of carbohydrate meal, despite different meal insulin boluses (8.1 [7.3–9.1] vs. 9.2 [8.2–9.6] U; p = 0.03), the subsequent basal insulin infusion over 4-h was not different in adequate estimation compared to overestimation (4.2 [3.9–5.3] vs. 4.3 [3.1–5.4] U; p = 0.60) (Table 3). For seven patients, the insulin bolus was limited during overestimation due to a safety parameter implemented in the algorithm: the algorithm does not allow a meal bolus to be superior to 21% of the individual’s total daily dose of insulin. This limitation is a built-in feature meant to improve safety of the algorithm. For three of these seven patients, this limitation resulted in an identical bolus between adequate estimation and overestimation.

For the 45 g of carbohydrate meal, the insulin bolus was also different between adequate estimation and overestimation (4.8 [3.7–5.6] vs. 8.4 [7.2–10.4] U; p < 0.001) and the basal insulin infusion was reduced, although not significant, during overestimation compared to adequate estimation (5.7 [4.1–7.4] vs. 4.3 [2.8–5.2] U; p = 0.06). Figure 3 shows that the basal rate was mostly reduced between 2 and 3-h post-meal in overestimation. No patient reached the 21% limit for the meal bolus.

Total glucagon delivery (given as micro-boluses) was also similar in both the adequate estimation and overestimation for the 75 g of carbohydrate meal (2.9 [0–5.8] vs. 1.0 [0–4.5] mg; p = 0.91) and for the 45 g of carbohydrate meal (0 [0–4.8] vs. 0.5 [0–5.9] mg; p = 0.55). Most of the glucagon was administered in the second half (between 2.5 and 4-h) of the postprandial period in the 75 g of carbohydrate meal and a little sooner in the 45 g of carbohydrate meal (between 1.5 and 4-h) (Figs 2 and 3).

In the virtual *in silico* trials, the meal bolus overestimation in all 3 meal sizes (the 2 same as the clinically tested and an additional 105 g scenario) did not have an effect on the % of time <4.0 mmol/L. The mean glucose and the % of time between 4 and 10 mmol/L improved as the bolus state changed from underestimated, to adequate, to overestimated. Similarly, the percentage of time >10 mmol/L decreased with increasing bolus size, and the amount of glucagon infusion increased, as shown in Table 4. The overestimation of carbohydrate content in all meal sizes improved glycemic control, but introduced a few additional hypoglycemic events. The 45 g virtual experiment with overestimation exhibited three additional hypoglycemic events over the adequate bolus estimation; the 75 g and the 105 g virtual experiments both exhibited one additional hypoglycemic event during the postprandial period.

## Discussion

This study examined the impact of misclassification of a meal in the context of dual-hormone closed-loop delivery combined with a simplified semi-quantitative strategy for the meal insulin bolus based on meal categorization. The results of the clinical trials indicate that despite increasing meal insulin boluses, overestimation of the meal category did not result in increased time spent in hypoglycemia. However, a few late postprandial hypoglycemic episodes requiring treatment were observed with meal bolus overestimation. Virtual studies yielded the same conclusions and offered the possibility to explore consequences of meal underestimation. This study suggests that dual-hormone closed-loop delivery combined with a simplified meal bolus strategy using three meal categories can safely handle, in most cases, overestimation or underestimation of meal size leading respectively to larger or smaller insulin boluses.

To our knowledge, this is the first study examining the impact of meal size classification errors in the context of dual-hormone closed-loop delivery. Chase *et al*. have previously shown that whether the bolus was omitted, administered with the meal, before the meal, or overestimated (by 30%), the safety of the single-hormone closed-loop delivery was preserved, and postprandial hyperglycemia was reduced in the context of overestimated bolus with the closed-loop delivery^15^. A study conducted in children and adolescents also found that single-hormone closed-loop delivery could compensate for an underestimated meal bolus (75% of the dose needed to cover the carbohydrate content of the meal) and improve mean blood glucose compared to usual care, without increasing hypoglycemia risk^16^. In the present study, a reduced postprandial hyperglycemia without increasing time below 4.0 mmol/L in the case of meal bolus overestimation was observed.

Simplifying meal bolus calculation could possibly improve quality of life for patients with type 1 diabetes by lessening the burden related to the need of precise carbohydrate counting; yet, it is important not to degrade post-meal glucose control in the process. It is also crucial not to increase the risk of hypoglycemia, an important burden for patients with type 1 diabetes, which impedes glycemic control and impairs quality of life^17–19^. Considering real-life carbohydrate counting errors are around 20%^14^, it is to be expected that mismatches might occur between the meal category selected by patients and the category associated with the exact meal carbohydrate content. With the simplified meal bolus strategy, such a mismatch would result in a larger discrepancy in meal bolus due to the 30 g of carbohydrate increment between each category. Examining the impact of such errors on the risk of hypoglycemia was thus important to ensure potential safety of this approach. The algorithm used in the present study has a security limit implemented which prevents a meal bolus to be larger than 21% of the participant’s average total daily insulin dose. This limit may have played a role in the prevention of hypoglycemia, especially for large meals where the limit can be reached more rapidly. The same 21% limit for the insulin bolus was also implemented in the virtual trials. In the 75 g of carbohydrate meal in the present study, the bolus limit was reached in seven out of ten patients, with the bolus being identical for overestimation and adequate estimation in three patients. The mean boluses remained significantly different even though the magnitude of the difference was attenuated. This limit did not impact the bolus difference in the smaller meals with 45 g of carbohydrate. Reduced meal boluses compared to estimated boluses strictly based on carbohydrate content of the meals and insulin-to-carbohydrate ratios has been used previously in the context of DH-CLS glucose control and has led to decreased time in hypoglycemia and comparable mean sensor glucose for DH-CLS delivery compared to conventional therapy^4^.

Despite the insulin bolus differences for both studies (75 g and 45 g of carbohydrate meals), mean sensor glucose and time in target range did not differ during the 4-h postprandial period when the meal was properly categorized or overestimated. Yet, for all interventions, peak glucose was high (median peak glucose >13.0 mmol/L) and time spent in hyperglycemia (>10.0 mmol/L) was between 44 and 69%. This could possibly be explained, in part, by the elevated starting (pre-meal) glucose (between 7.3 and 9.0 mmol/L) which can be typically observed in the morning combined with the lower insulin sensitivity at the breakfast meal which makes it a more difficult meal to control^20,21^.

This study was conducted using dual-hormone closed-loop delivery to limit the hypoglycemia risk associated with overestimation. The glucagon delivery in the clinical trials was similar between adequately or overestimated meals, for both the large and the regular meals. Its infusion in the late postprandial period for both meals may, however, have played a role in the hypoglycemia prevention. Several studies are suggesting an added benefit of glucagon incorporation in closed-loop delivery^22–24^; yet subgroups of patients or some situations may benefit more from its addition. The use of a simplified meal bolus strategy is possibly best accompanied with glucagon to prevent hypoglycemia risk, especially during the late postprandial phase, but investigating this strategy in the context of single-hormone closed-loop delivery would be of interest.

With actual available technologies, fully automated closed-loop delivery with no meal bolus or announcement has been consistently associated with significant postprandial hyperglycemic excursions and late postprandial hypoglycemia^22,23,25^. On the other hand, with a hybrid closed-loop delivery with a carbohydrate-matched insulin bolus, postprandial glucose control remains suboptimal, mostly due to post-meal hyperglycemia^26^. In addition, the need for precise carbohydrate counting is a limitation of hybrid closed-loop delivery from the perspective of relieving the patients from daily tasks. A simplified meal bolus strategy has potential benefits: 1) evaluating the meal size in terms of carbohydrate categorization is simpler than the current carbohydrate counting strategy and 2) simplified partial bolus can still be individualized for each patient and for each meal or time period. This strategy has been shown to be promising in outpatient settings despite the need for adjustments to limit hypoglycemia risk^12^. A similar strategy is also used with dual-hormone closed-loop system by Russell *et al*.^27^. In their strategy, patients select a meal type (breakfast, lunch or dinner) and size (typical, more than usual, less than typical or a small bite), and a meal-priming bolus is administered. The merits of their strategy for glucose control have been demonstrated in two large automated outpatient trials in children and adults^1,27^. Again, the safety of such strategies in the context of misclassification is essential to improve glucose control adequately.

We acknowledge some limitations in this study. First, it was conducted on a single meal, at breakfast. Breakfast is typically more difficult to control and results would need to be validated in outpatient studies with various meals and conditions e.g. optimal pre-meal glucose value, different time of the day or macronutrient content, etc. Also, the C-Peptide response was not measured in the present study. Although some cases on residual C-Peptide response have been reported^28,29^, decline in endogenous insulin secretion is seen with increasing disease duration and the clinical impact of these detectable, but very low, C-Peptide values remain questionable. We are also unable to evaluate whether our conclusions can be generalized to other artificial pancreas systems, especially the single-hormone closed-loop delivery systems, as well as algorithms that are not integrating a maximum meal bolus dose. Yet, these results provide helpful insights on the potential of the dual-hormone closed-loop delivery to safely handle inadequately estimated meal boluses. It is unlikely, but it remains possible, that more hypoglycemic episodes would occur in the case of meal carbohydrate content size overestimation with larger sample size. A strength of this study is the complementary *in silico* testing which confirmed our findings from the clinical trials and provided the opportunity to test additional scenarios. Finally, considering the complexity of postprandial glucose excursions, combination of closed-loop delivery with another or a second adjunctive therapy, pramlintide for example or with upcoming ultra-rapid insulins (e.g. Faster insulin Aspart (FiAsp)) could be interesting to examine.

A simplified meal bolus strategy in the context of closed-loop delivery could improve glycemic control while attenuating the burden associated with carbohydrate counting. This study showed that erroneous estimation of meal insulin boluses did not increase the risk of hypoglycemia assessed by time <4.0 mmol/L with a simplified meal bolus strategy and dual-hormone closed-loop delivery. Outpatient automated trials are needed to fully validate the safety of this strategy.

## Study Design and Methods

### Study design

The first part of this study is a single-blind, randomized, two-way, cross-over study to examine the impact of overestimation of a meal insulin bolus in the context of dual-hormone closed-loop operation (insulin and glucagon) combined with a simplified semi-quantitative meal-size estimation in adults with type 1 diabetes. Two separate meals (75 g and 45 g of carbohydrate) were tested in clinical settings during two subsequent clinical trials registered as NCT02626936 (December 10, 2015) and NCT02798250 (June 14, 2016). The second part of this study involved *in silico* clinical trials to explore the effect of an underestimated, adequately estimated, and overestimated boluses with three different meal sizes (45 g, 75 g, and 105 g of carbohydrate). These trials mimicked the clinical experiments in duration and protocol. The *in silico* trials were conducted using the same dual-hormone dosing predictive algorithm that was used for the human clinical trials^30^. The algorithm is based on a fuzzy-supervised model-based predictive technique combined with extended Kalman filtering and a set of heuristic rules. The algorithm was initialized using body weight, daily insulin requirements and insulin-to-carbohydrate ratios. Glucagon delivery is based on heuristic logical rules employing estimates of plasma glucose concentrations and their trends as provided by the Kalman filter and is accompanied with suspension of insulin delivery and reduced aggressiveness of insulin dosing. The *in silico* trials were conducted using the Development Platform for Artificial Pancreas Algorithms system^31^.

### Study population

Participants were recruited and tested from January 2016 to March 2017 at the *Institut de Recherches Cliniques de Montréal (IRCM)*, Montréal, Canada. We included adults (≥18 years of age) with type 1 diabetes for >one year, using insulin pump therapy for at least three months and using carbohydrate counting for meal boluses. Participants with poor glucose control (glycated hemoglobin >10%), with clinically significant micro (e.g. gastroparesis) or macrovascular complications or using medication likely to affect the results interpretation (e.g., agents affecting gastric emptying) were excluded. Other exclusion criteria were applied as detailed in the clinical trial registry. Ten participants were recruited for each study (75 g and 45 g of carbohydrate meals). Patients were offered to participate to one or both studies and four patients participated to both experiments. The studies obtained ethical approval from the IRCM ethics committee and all participants provided informed written consent. All experiments were performed in accordance with relevant guidelines and regulations. The simulated trials were conducted with 15 virtual patients as described below.

### Study procedures

All participants first completed a screening visit to assess eligibility. This visit included a medical visit with an endocrinologist to confirm eligibility and included anthropometric measurements (height, weight, waist circumference), a blood draw (glycated hemoglobin), and examination of insulin therapy records (average prandial and basal doses, basal rates, insulin-to-carbohydrate ratios). Participants were instructed how to install and use the continuous glucose monitoring systems (Dexcom G4 Platinum, Dexcom) or they could also set an appointment with study personnel for installation. They were instructed to calibrate sensors at least twice, and maximum four times daily using capillary blood glucose values.

Interventions were undertaken one to five days after sensor insertion. Subjects were admitted at IRCM at 7:00 am and were asked to fast from the previous night (midnight). If a correction bolus or a hypoglycemia correction (food or carbohydrate intake) was performed by patients within the four hours preceding the test, the test was reported or delayed depending on the magnitude of treatment or correction. Upon arrival, patients’ pumps were substituted with the study pump and a second pump containing glucagon was installed. During the interventions, variable subcutaneous insulin (patient’s usual insulin: Lispro, Aspart or Glulisine) and glucagon (Eli Lilly) infusion rates were used to regulate postprandial glucose levels using two infusion pumps (Accu-Check Combo, Roche). Glucagon was reconstituted according to the manufacturer’s instructions. Capillary blood glucose was measured to calibrate the sensor. At 7:00 am, closed-loop delivery was initiated and a standardized breakfast (45 g or 75 g of carbohydrate) was served at 9:00 am. Subjects were offered a choice between two menus (Appendix A), but consumed the same meal on both interventions. During the intervention, patients were allowed to do sedentary activities only (reading, watching television, playing video games, etc.). They were also allowed coffee or tea, but their consumption had to be replicated during the second intervention. The intervention ended at 1:00 pm. Interventions were scheduled 0 to 14 days apart.

The glucose levels as measured by the real-time sensor were entered manually into the computer every 10 minutes. The pumps’ infusion rates were then changed manually, using a remote control device, based on the computer-generated recommendation from the predictive algorithm^30^. The simplified bolus strategy is based on the estimated meal size (regular, large or very large) and the closed-loop delivery system gives the remaining insulin needed based on the sensor readings. Prandial boluses are calculated as individualized insulin-to-carbohydrate ratios multiplied by a fixed carbohydrate factor depending on the category. A regular meal is defined as 30 to 60 g of carbohydrate, with a bolus for 35 g. A large meal is defined as 60 to 90 g of carbohydrate, with a bolus for 65 g. A very large meal is defined as more than 90 g, with a bolus for 95 g. Patients thus have to select a category for their meal rather than count the exact amount of carbohydrates. For the purpose of this study, the meal category was entered by study personnel based on the randomized sequence (adequate estimation or overestimation): for the 75 g of carbohydrate meal, a bolus for 65 g (adequate estimation: large meal) or 95 g (overestimation: very large meal) was provided and for the 45 g of carbohydrate meal, a bolus for 35 g (adequate estimation: regular meal) or 65 g (overestimation: large meal) was provided. Participants were blinded to the intervention but not study personnel. To test the effect of overestimation on very large meals, we simulated *in silico* an additional trial with a 105 g of carbohydrate meal with a bolus for 65 g (underestimation: large meal), a bolus for 95 g (adequate estimation: very large meal), and a bolus for 125 g (overestimation: overbolus, undefined meal category). Insulin boluses were administered 10 minutes before the beginning of the meal. A balanced randomization, created by a third-party unrelated to the project was used to determine the order of the interventions. Randomization allocation was preserved in sealed envelopes opened by the study personnel following admission.

### Simulated Trials

To augment the clinical trials in exploring the impact of meal bolus overestimation using the simplified semi-quantitative strategy within a closed-loop system, we used the cloud-based Development Platform for Artificial Pancreas Algorithms^31^ system to conduct the simulated clinical trials. The system was connected with the same predictive algorithm^30^ used in the trials and followed the same clinical protocols (4-hour post-meal period, meals at 9 am, and dosing amounts calculated every 10 minutes). The simulated trials were performed on 15 virtual subjects whose glucoregulatory responses were modeled from the responses of real type 1 diabetes patients^24^. The patient model incorporates inter- and intra-individual variability in patient’s glucose, insulin, and glucagon dynamics.

### Outcomes

The primary endpoint was the percentage of time with sensor glucose below 4.0 mmol/L for the 4-hour period following the meal compared between dual-hormone closed-loop delivery with adequate estimation (proper categorization) vs. dual-hormone closed-loop delivery with overestimation (erroneous categorization). Secondary endpoints were also calculated for the 4-hour period following the meal and included; mean sensor glucose, percentage of time for which sensor glucose was in the target range (4.00–10.00 mmol/L), percentage of time spent above the target range, peak and time to peak sensor glucose, insulin bolus, total amount of insulin delivered, and the total amount of glucagon delivered.

### Statistical analyses

The effect of the interventions on the continuous outcomes was estimated using a multivariate linear mixed effect model (LMEM) with the intervention (2 levels), treatment sequence, period, and starting glucose level entered as fixed effects, and subject nested within sequence as random effect. If highly skewed (P value for Shapiro–Wilk test < 0.05), outcomes were transformed using an appropriate Box-Cox transformation prior to modeling the linear mixed model. This is an exploratory study and power calculations were not performed. Data are presented as median [Interquartile Range].

All data generated or analysed during this study are included in this published article (and its Supplementary Information files).

### Clinical trial registry numbers

NCT02626936 (December 10, 2015), NCT02798250 (June 14, 2016).

## Electronic supplementary material

Supplementary Information
