# Supplementary material for: Impact of erroneous meal insulin bolus with dual-hormone artificial pancreas using a simplified bolus strategy - A randomized controlled trial
Source: Sci Rep. 2018 Feb 8;8:2621. doi: 10.1038/s41598-018-20785-4 (PMC5805693; doi:10.1038/s41598-018-20785-4)
Supplement: Supplementary file 1 — Supplementary Information [file 41598_2018_20785_MOESM1_ESM.pdf]

Impact of erroneous meal insulin bolus with dual-hormone artificial pancreas using a simplified bolus strategy - A randomized controlled trial
(SREP-17-32041B)

¥ Véronique Gingras[1-2]. ¥ Mohamed Raef Smaoui[1-2]. Charlotte Cameli[1-3]. Virginie Messier[1]. Martin Ladouceur[4]. Laurent Legault[5]. Rémi Rabasa-Lhoret[1-3.6] *

SUPPLEMENTARY INFORMATION: MENU AND MEAL COMPOSITION IN THE CLINICAL TRIALS

Menu and meal composition for the 75g of carbohydrates meal

OPTION A									
**Qty**	**Weight or volume**	**Food item**	**Brand**	**Kcal**	**Total Carbohydrates (g)**	**Dietary Fibres (g)**	**Digestible Carbohydrates (g)**	**Proteins (g)**	**Lipids (g)**
2	25 g	Whole wheat bread slices	St-Méthode Club	180	31	7	24	9	2.5
1	30 g	Creamy regular peanut butter	Kraft	180	8	2	6	6	16
1	15 ml	Raspberry jam	Double Fruit	40	10	0	10	0.1	0
1	100 g	Greek vanilla yogurt	Oikos	100	13	0	13	8	1.5
1	200 ml	Pure orange juice	Oasis	88	21	0	21	0.8	0
			**TOTAL**	**588**	**83**	**9**	**74**	**23.9**	**20**

OPTION B									
**Qty**	**Weight or volume**	**Food item**	**Brand**	**Kcal**	**Total Carbohydrates (g)**	**Dietary Fibres (g)**	**Digestible Carbohydrates (g)**	**Proteins (g)**	**Lipids (g)**
1	375 ml	Cheerios multigrain cereals	General Mills	165	36	4.5	32	3	2.3
1	200 ml	Milk, 2% fat	Québon	100	10	0	10	7	4
1	100 g	Raspberry yogurt 2.9% fat	Activia	97	14	0	14	4	2.9
1	20 g	Very mild cheddar cheese	P'tit Québec	80	0	0	0	5	7
1	107 ml	Diced peaches in juice	Dole	80	19	1	18	1	0
			**TOTAL**	**522**	**79**	**5.5**	**74**	**20**	**16.2**

Menu and meal composition for the 45g of carbohydrates meal

OPTION A									
**Qty**	**Weight or volume**	**Food item**	**Brand**	**Kcal**	**Total Carbohydrates (g)**	**Dietary Fibres (g)**	**Digestible Carbohydrates (g)**	**Proteins (g)**	**Lipids (g)**
1	25 g	Whole wheat bread slices	St-Méthode Club	90	15.5	3.5	12	4.5	1.25
1	15g	Creamy regular peanut butter	Kraft	90	4	1	3	3	8
1	7.5 ml	Raspberry jam	Double Fruit	20	5	0	5	0	0
1	100 g	Greek vanilla yogurt	Oikos	100	13	0	13	8	1.5
1	20 g	Unsweetened apple sauce	Mott's Fruitsa-tions	80	0	0	0	5	7
			**TOTAL**	**430**	**49.5**	**5.5**	**44**	**20.5**	**17.75**

OPTION B									
**Qty**	**Weight or volume**	**Food item**	**Brand**	**Kcal**	**Total Carbohydrates (g)**	**Dietary Fibres (g)**	**Digestible Carbohydrates (g)**	**Proteins (g)**	**Lipids (g)**
1	175 ml	Cheerios multigrain cereals	General Mills	110	23	2	21	2	2
1	200 ml	Milk, 2% fat	Québon	97	7	0	7	9	4
1	100 g	Raspberry yogurt 2.9% fat	Activia	100	14	0	14	4	3
1	20 g	Very mild cheddar cheese	P'tit Québec	80	0	0	0	5	7
			**TOTAL**	**387**	**47**	**2**	**45**	**18**	**15.9**
